# Genome Sequence of a Foot-and-Mouth Disease Virus Detected in Indonesia in 2022

**DOI:** 10.1128/mra.01081-22

**Published:** 2023-01-09

**Authors:** Nuryani Zainuddin, Edy Budi Susila, Hendra Wibawa, Rosmalina Sari Dewi Daulay, Putriani Endah Wijayanti, Dini Fitriani, Dewi Noor Hidayati, Syafrison Idris, Jemma Wadsworth, Noemi Polo, Hayley M. Hicks, Valerie Mioulet, Nick J. Knowles, Donald P. King

**Affiliations:** a Directorate General of Livestock and Animal Health Services, Ministry of Agriculture of Indonesia, Jakarta, Indonesia; b Pusat Veteriner Farma, National Center for Veterinary Biologics, Surabaya, Indonesia; c Balai Besar Veteriner Wates, Disease Investigation Center, Wates, Yogyakarta, Indonesia; d The Pirbright Institute, Pirbright, United Kingdom; DOE Joint Genome Institute

## Abstract

During 2022, outbreaks of foot-and-mouth disease (FMD) were reported across the islands of Indonesia, a country that had previously maintained an FMD-free (without vaccination) status since 1990. This report describes the near-complete genome sequence of a representative FMD virus collected from these cases belonging to the O/ME-SA/Ind-2001e lineage.

## ANNOUNCEMENT

Foot-and-mouth disease (FMD) is a transboundary disease that affects domesticated cloven-hooved livestock and wildlife. The causative virus (FMD virus [FMDV]; family *Picornaviridae*, genus *Aphthovirus*) can easily spread to cause outbreaks in new geographical locations. Since May 2022, FMD outbreaks have been reported in Indonesia, a country that had not experienced an FMD outbreak since 1983. This report describes the near-complete genome sequence of an FMDV (O/ISA/1/2022) that was present in a vesicular epithelium sample collected on 4 May 2022 from cattle in the Lamongan District of East Java Province. Total RNA was extracted from a 10% tissue suspension using an RNeasy minikit (Qiagen), and first-strand cDNA synthesis (reverse transcription) was performed using the Superscript III first-strand synthesis system (Life Technologies) as previously described (1). Second-strand synthesis was undertaken using 20 μL of cDNA with a second-strand synthesis kit (New England Biolabs [NEB]). One nanogram of the double-stranded DNA sample was used to prepare sequencing libraries using the Nextera XT DNA sample preparation kit (Illumina). All kits were used according to the manufacturer’s instructions, and the sequencing libraries were analyzed on a MiSeq system (Illumina) as previously described (1). A paired-end sequencing run of 2 × 150-nucleotide (nt) read lengths generated 2,761,530 reads. The resulting sequences were mapped against the genome sequence of strain O/SKR/1/2017 (GenBank accession no. MG983730) using SeqMan NGen software with default quality trimming settings and were visualized using SeqMan Pro (Lasergene package v16; DNAStar, Inc.). Mapping resulted in 112,046 reads (8,208 nt; G+C content, 54%; median coverage, 5,995×); 5 nucleotides at the 5′ end of the genome were not determined, but a short region of the 3′ poly(A) tail (33 nt) was sequenced. FMDV genomes contain a long poly(C) tract (usually about 70 to 250 bases) within their 5′ untranslated region (UTR) (2), which was not sequenced; instead, an artificial poly(C) tract consisting of 11 Cs was inserted at position 364. A single open reading frame of 6,999 nt was predicted using BioEdit v7.2.5 (3) to encode a polyprotein of 2,333 amino acids containing 4 structural and 10 nonstructural proteins.

**FIG 1:**
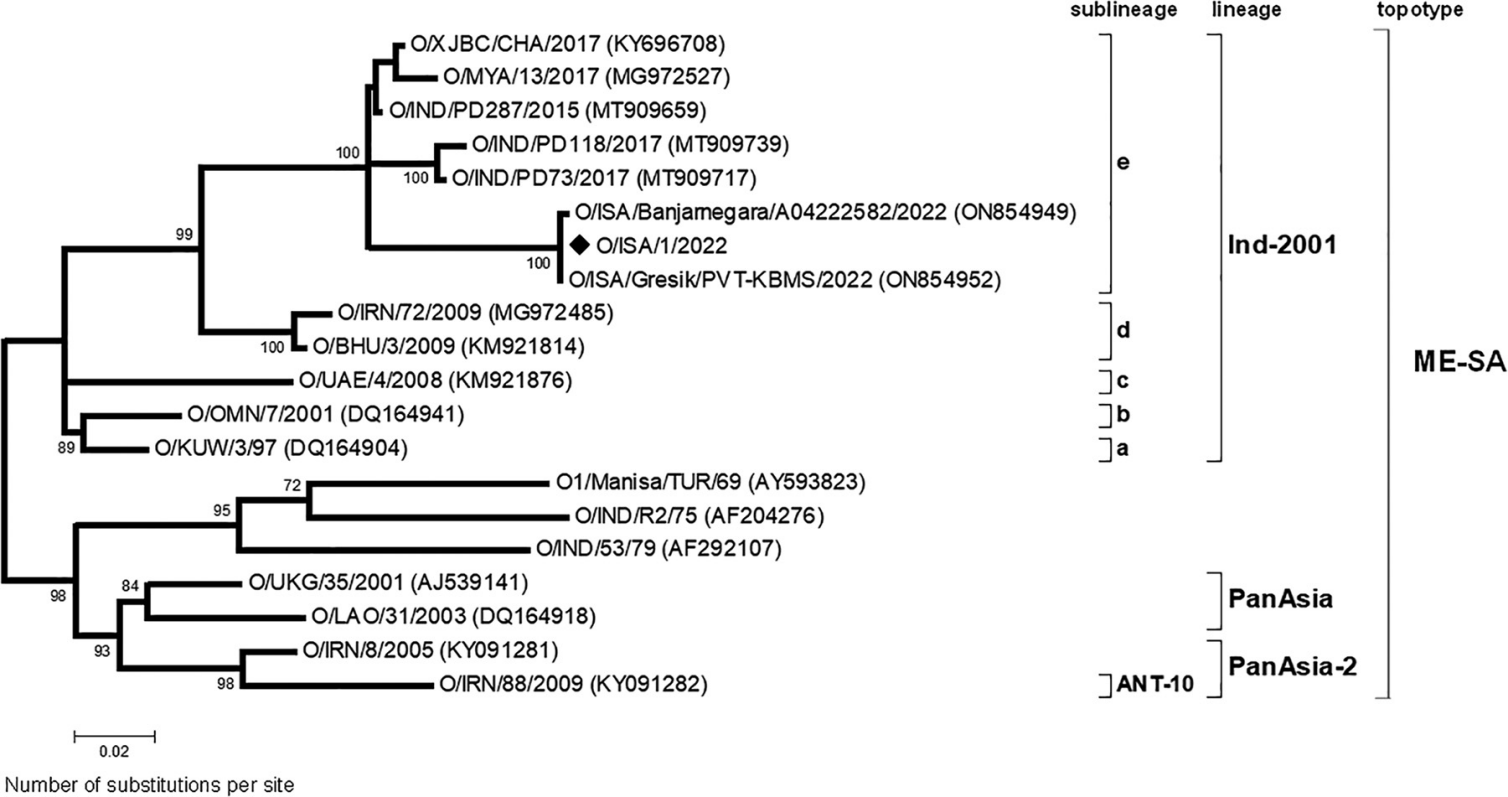
Midpoint-rooted maximum likelihood tree of the VP1-coding region. The tree was produced using MEGA7 (4) using a multiple sequence alignment that was built using the ClustalW function within BioEdit (3). The evolutionary history was inferred based on the Tamura 3-parameter model. The rate variation model allowed for some sites to be evolutionarily invariable ([+I]; 65.88% sites). The percentage of trees in which the associated taxa clustered together (by bootstrap analysis) is shown next to the branches (only values of 70% and above are shown). The tree shows representative reference viruses representing lineages and sublineages within the Middle East-South Asia (ME-SA) topotype. The Indonesian sequence reported in this study is indicated by a black diamond.

Phylogenetic analyses (Fig. 1) of the VP1-coding region characterized the sequence as a member of the O/ME-SA/Ind-2001e lineage, sharing the closest nucleotide identity to other contemporary sequences collected from Indonesia. Related viruses have recently spread from South Asia (Bangladesh, Bhutan, India, Nepal, and Sri Lanka) into East Asia (China, South Korea, Russia, and Mongolia), mainland Southeast Asia (Cambodia, Laos, Malaysia, Myanmar, Thailand, and Vietnam), and Central Asia (Russia and Kazakhstan) (5–9). This coding-complete genome sequence can be used to confirm the suitability of molecular assays that are being used to detect O/ME-SA/Ind-2001e viral RNA in clinical samples; the sequence will also provide a starting point for further high-resolution analyses (10, 11) that will help us to understand the origin and spread of this lineage across the Indonesian archipelago.

### Data availability.

The nucleotide sequence of FMDV O/ISA/1/2022 has been deposited at GenBank under the accession no. OP585403. The raw sequence data were deposited in the NCBI Sequence Read Archive under BioProject accession no. PRJNA879345.
